# Correction: Early childhood education and care quality and associations with child outcomes: A meta-analysis

**DOI:** 10.1371/journal.pone.0293056

**Published:** 2023-10-12

**Authors:** Antje von Suchodoletz, D. Susie Lee, Junita Henry, Supriya Tamang, Bharathy Premachandra, Hirokazu Yoshikawa

There are errors in the Funding section. The correct Funding statement is: Specific grant number: GST03 Initials of authors who received award: AvS Specific grant number: EDU/0500057415 Initials of authors who received award: AvS, HY

Full names of commercial companies that funded the study or authors: no commercial company funded the study or authors.

Work preparatory to this meta-analysis was funded by the Organization for Economic Cooperation and Development (Contract Reference: EDU/0500057415).

The systematic literature search and the coding for this meta-analysis was funded by the Global TIES for Children Research Center at New York University Abu Dhabi (GST03).

URL to sponsor’ website: https://nyuad.nyu.edu/en/research/faculty-labs-and-projects/global-ties-for-children.html

The funders (other than the named authors) had no role in study design, data collection and analysis, decision to publish, or preparation of the manuscript.
