# Supplementary material for: Early childhood education and care quality and associations with child outcomes: A meta-analysis
Source: PLoS One. 2023 May 25;18(5):e0285985. doi: 10.1371/journal.pone.0285985 (PMC10212181; doi:10.1371/journal.pone.0285985)
Supplement: S1 File — (DOCX) [file pone.0285985.s003.docx]

Early Childhood Education and Care Quality and Associations with Child Outcomes: A Meta-Analysis

Supporting Information (SI) 1

List of Keywords for Literature Search

***Search-String ECE* [AND]**

“preprimary” OR “pre-primary” OR “pre primary” OR “early childhood education” OR “early child care” OR “early education” OR “early education and care” OR “preschool education” OR “pre school” OR “pre-k*” OR “preschool” OR “kindergarten” OR “pre-school” OR “pre-K” OR “pre-kindergarten” OR “prekindergarten”

***Search-String Process Quality* [AND]**

“quality” OR “classroom quality” OR “child care quality” OR “process quality” OR “program quality” OR “programme quality” OR “interaction quality” OR “instructional quality” OR “climate” OR “teaching approaches” OR “teaching practices” OR “teaching strategies” OR “instruction”

***Search-String Structural Characteristics* [AND]**

“structural characteristics” OR “structural features” OR “structure” OR “teacher” OR “classroom” OR “program” OR “classroom characteristics” OR “program characteristics” OR “classroom features” OR “program features”

***Search-String Child Outcomes***

“school readiness” OR “child outcomes” OR “child development” OR “pre-academic achievement” OR “pre-academic outcomes” OR “pre-academic skills” OR “academic achievement” OR “academic outcomes” OR “academic skills” OR “language” OR “literacy” OR “vocabulary” OR “language development” OR “literacy development” OR “vocabulary development” OR “language outcomes” OR “literacy outcomes” OR “vocabulary outcomes” OR “language skills” OR “literacy skills” OR “vocabulary skills” OR “math” OR “numeracy” OR “math development” OR “numeracy development” OR “math outcomes” OR “numeracy outcomes” OR “math skills” OR “numeracy skills” OR “social outcomes” OR “outcomes” OR “skills” OR “development” OR “social skills” OR “social development” OR “socioemotional” OR “socio-emotional” OR “socioemotional outcomes” OR “socioemotional skills” OR “socioemotional development” OR “socio-emotional outcomes” OR “socio-emotional skills” OR “socio-emotional development” OR “behavioral outcomes” OR “behavioral skills” OR “behavioral development” OR “prosocial behavior” OR “prosocial skills” OR “aggressive behavior” OR “aggressive behavior” OR “aggressive behaviour” OR “aggressive behaviour” OR “aggressive problems” OR “self-regulation” OR “self regulation”

We searched the databases PsychInfo, ERIC, Pubmed, and EbscoHost. For each of the databases we ran the above keyword search with separate title, keywords, and abstract filters for each search-string.
