# Supplementary material for: Early childhood education and care quality and associations with child outcomes: A meta-analysis
Source: PLoS One. 2023 May 25;18(5):e0285985. doi: 10.1371/journal.pone.0285985 (PMC10212181; doi:10.1371/journal.pone.0285985)
Supplement: S3 File — (DOCX) [file pone.0285985.s005.docx]

Early Childhood Education and Care Quality and Associations with Child Outcomes: A Meta-Analysis

Supporting Information (SI) 3

Funnel Plots


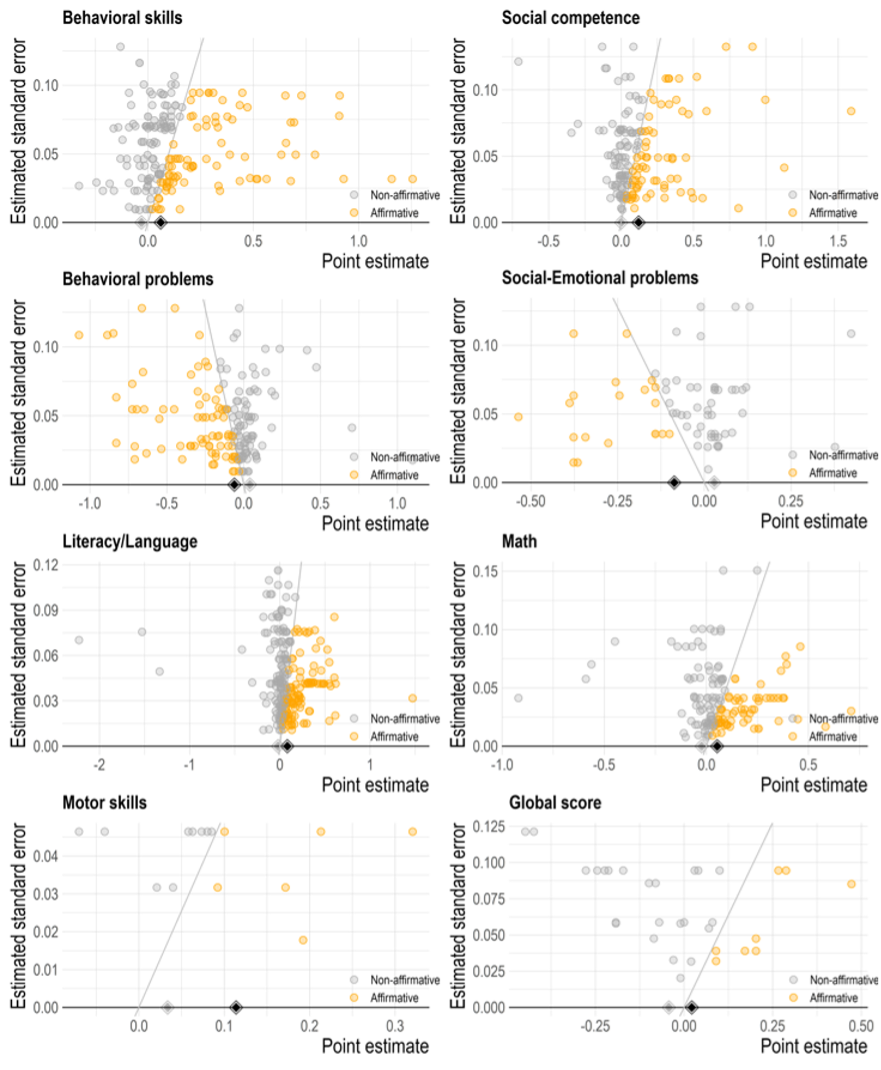


*Figure S1.* Significance funnel plots for ECEC quality-child outcome associations. Point estimates are Fisher’s *z*-scale. Estimates lying on the diagonal line had *p*=0.05. Estimates were distinguished by whether they were ‘affirmative’ (i.e., *p*-value less than 0.05) or ‘non-affirmative’. Grey diamonds are robust clustered estimates among non-affirmative effect sizes only, and black diamonds are robust clustered estimates across all effect sizes.
