# Supplementary material for: Early childhood education and care quality and associations with child outcomes: A meta-analysis
Source: PLoS One. 2023 May 25;18(5):e0285985. doi: 10.1371/journal.pone.0285985 (PMC10212181; doi:10.1371/journal.pone.0285985)
Supplement: S4 File — (DOCX) [file pone.0285985.s006.docx]

Early Childhood Education and Care Quality and Associations with Child Outcomes: A Meta-Analysis

Supporting Information (SI) 4

Sensitivity Analysis

**Type of effect size measure**

We explored whether differences in the type of effect size measure (zero-order correlation versus regression coefficient) give rise to different or similar associations between process quality indicators and child outcomes. The comparison indicated that effect sizes reported as zero-order correlation coefficient were, on average, 0.05 higher than effect sizes reported as regression coefficients. However, the difference was not big enough to conclude that the two types of coefficients were significantly different (Kruskal-Wallis chi-squared = 0.00, df = 1, *p*-value = 0.97). Figure S2 summarizes the results.


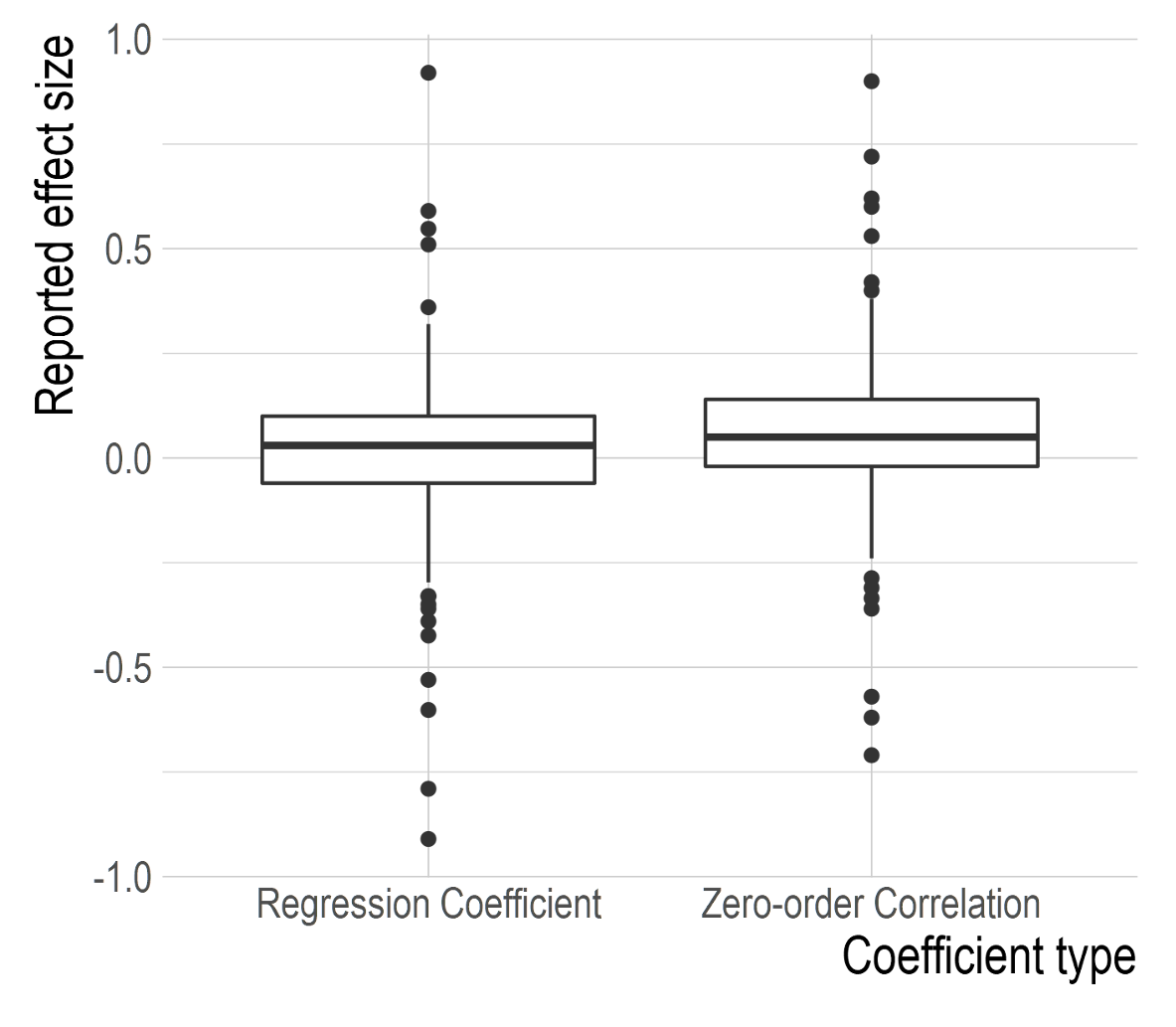


*Figure S2*. Exploring differences in the associations between process quality and child outcomes by the type of effect size measure (regression coefficient versus zero-order correlation coefficient).
