# Supplementary material for: Early childhood education and care quality and associations with child outcomes: A meta-analysis
Source: PLoS One. 2023 May 25;18(5):e0285985. doi: 10.1371/journal.pone.0285985 (PMC10212181; doi:10.1371/journal.pone.0285985)
Supplement: S5 File — (DOCX) [file pone.0285985.s007.docx]

Early Childhood Education and Care Quality and Associations with Child Outcomes: A Meta-Analysis

Supporting Information (SI) 5

Association Between ECEC Quality and Child Outcomes (Only Studies from High-Income Countries)

Most child outcome categories showed a significant overall association with ECEC quality when only high-income countries were included in the analysis (in the description of results below, n refers to the number of unique studies). In high-income countries, higher levels of ECEC quality were significantly related to higher levels of academic outcomes (literacy, n=85: 0.06, 95% C.I. 0.00 – 0.13; math, n=48: 0.06, 95% C.I. 0.04 – 0.10), behavioral skills (n=54: 0.12, 95% C.I. 0.07 – 0.17), and social competence (n=50: 0.11, 95% C.I. 0.06 – 0.17), and lower levels of behavioral (n=56: -0.14, 95% C.I. -0.20 - -0.08) and social-emotional problems (n=25: -0.07, 95% C.I. -0.13 - -0.01). Motor skills and global assessment of child outcomes were not included in the analyses because not enough unique studies were available.
