# Supplementary material for: Early childhood education and care quality and associations with child outcomes: A meta-analysis
Source: PLoS One. 2023 May 25;18(5):e0285985. doi: 10.1371/journal.pone.0285985 (PMC10212181; doi:10.1371/journal.pone.0285985)
Supplement: S6 File — (DOCX) [file pone.0285985.s008.docx]

Early Childhood Education and Care Quality and Associations with Child Outcomes: A Meta-Analysis

Supporting Information (SI) 6

Associations Between Structural Characteristics and Child Outcomes

The associations between structural characteristics and child outcomes were not significant (literacy, n=28: 0.03, 95% C.I. -0.02 – 0.08; math, n=16: 0.01, 95% C.I. -0.04 – 0.05; behavioral skills, n=9: 0.01, 95% C.I. -0.04 – 0.05; social competence, n=13: 0.03, 95% C.I. -0.03 – 0.08; behavioral problems, n=13: -0.03, 95% C.I. -0.07 - 0.13; social-emotional problems, n=2: -0.02, 95% C.I. -0.88 - 0.84; motor skills, n=2: 0.14, 95% C.I. -0.61 – 0.89; and global assessment of child outcomes, n=5: -0.05, 95% C.I. -0.26 – 0.15).
