# Supplementary material for: Early childhood education and care quality and associations with child outcomes: A meta-analysis
Source: PLoS One. 2023 May 25;18(5):e0285985. doi: 10.1371/journal.pone.0285985 (PMC10212181; doi:10.1371/journal.pone.0285985)
Supplement: S7 File — (DOCX) [file pone.0285985.s009.docx]

Early Childhood Education and Care Quality and Associations with Child Outcomes: A Meta-Analysis

Supporting Information (SI) 7

Differences in ECEC Quality – Child Outcome Associations by the Type of Process Quality Domain


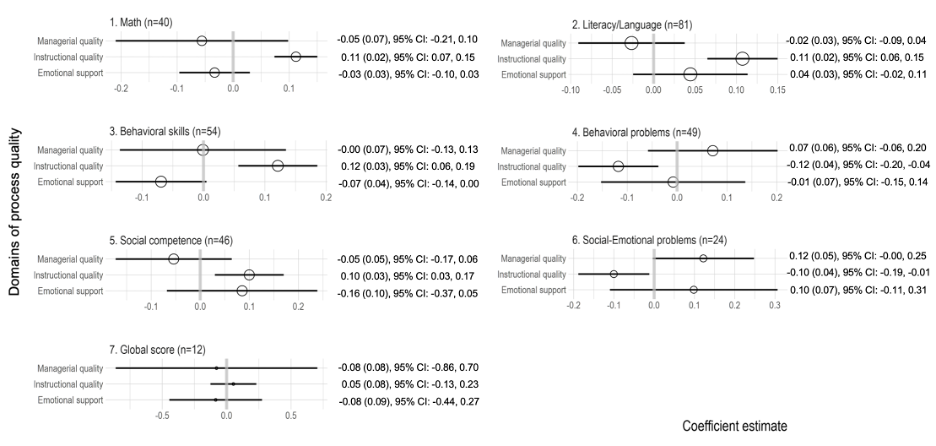


*Figure S3*. Associations between ECEC process quality and child outcomes by process quality domains (number of unique studies is given in the parentheses). Process quality domains were grouped into instructional, emotional, and managerial aspects. The estimates’ 95% confidence intervals are shown in lines. Control variables were not included.
