# Supplementary material for: Early childhood education and care quality and associations with child outcomes: A meta-analysis
Source: PLoS One. 2023 May 25;18(5):e0285985. doi: 10.1371/journal.pone.0285985 (PMC10212181; doi:10.1371/journal.pone.0285985)
Supplement: S8 File — (DOCX) [file pone.0285985.s010.docx]

Early Childhood Education and Care Quality and Associations with Child Outcomes: A Meta-Analysis

Supporting Information (SI) 8

Differences in Effect Size by Process Quality Domain

Table S2

*Differences in effect size by process quality domain*

| Child Outcome | Included studies (*n*) | Coefficient (SE) | | *t* (df) | | 95% CI  lower, upper |
| --- | --- | --- | --- | --- | --- | --- |
| Math | 40  40 | -0.06  -0.03 | (0.07)  (0.03) | -0.79  -0.98 | (11.83)  (25.34) | -0.22, 0.10  -0.10, 0.04 |
|  | 21 | 0.03 | (0.07) | 0.42 | (12.58) | -0.13, 0.19 |
| Language/Literacy | 81  81 | -0.03  0.05 | (0.03)  (0.04) | -0.88  1.27 | (26.21)  (61.51) | -0.09, 0.04  -0.03, 0.12 |
|  | 51 | 0.06 | (0.04) | 1.69 | (27.06) | -0.01, 0.13 |
| Behavioral skills | 54  54 | 0.00  -0.06 | (0.06)  (0.04) | 0.06  -1.62 | (22.47)  (29.48) | -0.13, 0.14  -0.15, 0.01 |
|  | 30 | -0.05 | (0.04) | -1.10 | (25.97) | -0.14, 0.04 |
| Social competence | 49  49 | -0.06  0.06 | (0.05)  (0.08) | -1.16  0.85 | (11.60)  (13.52) | -0.18, 0.05  -0.10, 0.24 |
|  | 16 | 0.15 | (0.07) | 1.97 | (11.59) | -0.02, 0.31 |
| Behavioral problems | 49  49 | 0.08  0.03 | (0.05)  (0.05) | 1.53  0.06 | (9.90)  (10.43) | -0.04, 0.21  -0.11, 0.12 |
|  | 12 | -0.09 | (0.05) | -1.61 | (9.89) | -0.20, 0.03 |
| Social-emotional problems | 24  24 | 0.10  0.08 | (0.05)  (0.06) | 2.26  1.22 | (6.97)  (3.31) | -0.00, 0.21  -0.12, 0.28 |
|  | 7 | -0.01 | (0.04) | -0.24 | (3.45) | -0.14, 0.12 |
| Global Score | 12  12 | -0.07  0.03 | (0.11)  (0.12) | -0.64  0.24 | (1.15)  (2.52) | -1.15, 1.00  -0.38, 0.44 |
|  | 3 | -0.08 | (0.18) | -0.46 | (1.00) | -2.34, 2.18 |

*Note*. The statistics reported in the first row for each outcome reflect managerial quality in reference to instructional quality; the statistics reported in the second row for each outcome reflect emotional quality in reference to instructional quality; the statistics reported in the third row for each outcome reflect emotional quality in reference to managerial quality.

The analyses controlled for sex composition (proportion of girls in the sample) and average child age in the sample (in months).
