# Supplementary material for: Early childhood education and care quality and associations with child outcomes: A meta-analysis
Source: PLoS One. 2023 May 25;18(5):e0285985. doi: 10.1371/journal.pone.0285985 (PMC10212181; doi:10.1371/journal.pone.0285985)
Supplement: S9 File — (DOCX) [file pone.0285985.s011.docx]

Early Childhood Education and Care Quality and Associations with Child Outcomes: A Meta-Analysis

Supporting Information (SI) 9

Tests of Structural Indicators of ECEC Quality as Moderators of Process Quality-Child Outcome Associations

Table S3

*Tests of structural indicators of ECEC quality as moderators of process quality-child outcome associations*

| Child Outcome | Structural indicator | Included studies (*n*) | Coefficient (SE) | | *t* (df) | | 95% CI  lower, upper |
| --- | --- | --- | --- | --- | --- | --- | --- |
| Math | Teacher age | 5 | -0.00  -0.03 | (0.01)  (0.00) | -0.05  -8.65 | (2.25)  (1.00) | -0.04, 0.04  -0.06, 0.01 |
|  | Years of experience | 13 | -0.02  -0.02 | (0.02)  (0.02) | -1.46  -1.43 | (4.19)  (4.13) | -0.07, 0.02  -0.07, 0.02 |
|  | Teacher education | 21 | -0.02  0,01 | (0.05)  (0.05) | -0.46  0.26 | (11.18)  (6.94) | -0.12, 0.08  -0.11, 0.14 |
|  | Group size | 13 | -0.02  -0.02 | (0.02)  (0.02) | -1.46  -1.43 | (4.19)  (4.13) | -0.07, 0.02  -0.07, 0.02 |
|  | Teacher-child ratio^a^ | 10 | 0.01 | (0.01) | 1.28 | (1.58) | -0.04, 0.07 |
| Language/Literacy | Teacher age^a^ | 14 | -0.02 | (0.01) | -1.57 | (1.83) | -0.08, 0.04 |
|  | Years of experience | 43 | -0.00  -0.00 | (0.01)  (0.01) | -0.24  -0.28 | (15.42)  (15.90) | -0.02, 0.02  -0.02, 0.02 |
|  | Teacher education | 52 | -0.12  -0.12 | (0.04)  (0.04) | -3.45  -2.85 | (30.11)  (24.27) | -0.20, -0.05  -0.21, -0.03 |
|  | Group size | 43 | -0.00  -0.00 | (0.01)  (0.01) | -0.24  -0.28 | (15.42)  (15.90) | -0.02, 0.02  -0.02, 0.02 |
|  | Teacher-child ratio^a^ | 18 | 0.01 | (0.00) | 3.37 | (2.20) | -0.00, 0.02 |
| Behavioral skills | Teacher age^a^ | 15 | -0.01  -0.01 | (0.01)  (0.01) | -0.94  -0.83 | (1.91)  (2.03) | -0.06, 0.04  -0.06, 0.04 |
|  | Years of experience | 24 | -0.00  -0.01 | (0.01)  (0.01) | -0.71  -1.23 | (5.25)  (5.63) | -0.02, 0.01  -0.02, 0.01 |
|  | Teacher education | 30 | 0.05  0.08 | (0.06)  (0.09) | 0.92  0.89 | (13.50)  (5.00) | -0.07, 0.18  -0.16, 0.32 |
|  | Group size | 24 | -0.00  -0.00 | (0.01)  (0.01) | -0.71  -1.23 | (5.25)  (5.63) | -0.02, 0.01  -0.02, 0.01 |
|  | Teacher-child ratio^a^ | 9 | 0.01 | (0.01) | 1.43 | (2.64) | -0.01, 0.03 |
| Social competence | Teacher age^a^ | 12 | -0.01  -0.01 | (0.00)  (0.01) | -2.05  -1.77 | (3.12)  (3.06) | -0.02, 0.00  -0.03, 0.01 |
|  | Years of experience | 21 | -0.01  -0.01 | (0.01)  (0.01) | -1.16  -1.17 | (7.26)  (6.90) | -0.03, 0.01  -0.03, 0.01 |
|  | Teacher education | 21 | 0.02  0.01 | (0.06)  (0.08) | 0.30  0.18 | (6.54)  (5.77) | -0.12, 0.16  -0.19, 0.22 |
|  | Group size | 21 | -0.01  -0.01 | (0.01)  (0.01) | -1.16  -1.17 | (7.26)  (6.91) | -0.03, 0.01  -0.03, 0.01 |
|  | Teacher-child ratio^a^ | 9 | -0.00 | (0.01) | -0.33 | (1.91) | -0.06, 0.05 |
| Behavioral problems | Teacher age^a^ | 11 | 0.00  0.01 | (0.01)  (0.01) | 0.25  0.80 | (2.91)  (2.26) | -0.03, 0.03  -0.03, 0.04 |
|  | Years of experience | 24 | 0.00  0.01 | (0.01)  (0.01) | 0.41  0.67 | (6.56)  (6.21) | -0.01, 0.02  -0.01, 0.03 |
|  | Teacher education | 10 | -0.08  -0.08 | (0.08)  (0.09) | -0.88  -0.89 | (10.70)  (5.93) | -0.28, 0.12  -0.29, 0.14 |
|  | Group size | 24 | 0.00  0.01 | (0.01)  (0.01) | 0.41  0.67 | (6.56)  (6.21) | -0.01, 0.02  -0.01, 0.03 |
|  | Teacher-child ratio^a^ | 11 | 0.02 | (0.00) | 7.42 | (2.50) | 0.01, 0.03 |
| Social-emotional problems | Teacher age^a^ | 6 | -0.00  -0.01 | (0.01)  (0.01) | -0.60  -0.66 | (1.79)  (1.30) | -0.05, 0.04  -0.11, 0.09 |
|  | Years of experience | 10 | 0.01  0.00 | (0.00)  (0.00) | 1.41  1.42 | (2.29)  (2.35) | -0.01, 0.02  -0.01, 0.01 |
|  | Teacher education | 7 | 0.12  0.05 | (0.11)  (0.04) | 1.13  1.33 | (1.77)  (1.81) | -0.40, 0.64  -0.13, 0.23 |
|  | Group size | 10 | 0.01  0.00 | (0.00)  (0.00) | 1.41  1.42 | (2.29)  (2.35) | -0.01, 0.02  0.01, 0.01 |
|  | Teacher-child ratio^b^ |  |  |  |  |  |  |

*Note*. The statistics reported in the first row for each outcome reflect results without control variables; the statistics reported in the second row for each outcome reflect results with control variables (sex composition: proportion of girls in the sample and average child age in the sample, in months).

^a^Information regarding control variables was not available. ^b^No studies available to test the moderation.
