# Supplementary material for: Early childhood education and care quality and associations with child outcomes: A meta-analysis
Source: PLoS One. 2023 May 25;18(5):e0285985. doi: 10.1371/journal.pone.0285985 (PMC10212181; doi:10.1371/journal.pone.0285985)
Supplement: S10 File — (DOCX) [file pone.0285985.s012.docx]

Early Childhood Education and Care Quality and Associations with Child Outcomes: A Meta-Analysis

Supporting Information (SI) 10

Tests of Process Quality Indicators of ECEC Quality as Moderators of Structural Quality-Child Outcome Associations

Table S4

*Tests of process quality indicators of ECEC quality as moderators of structural quality-child outcome associations*

| Child Outcome | Process indicator | Included studies (*n*) | Coefficient (SE) | | *t* (df) | | 95% CI  lower, upper |
| --- | --- | --- | --- | --- | --- | --- | --- |
| Math | Emotional support | 4 | 0.13  -0.99 | (0.10)  (--) | 1.25  -- | (1.00)  (1.76) | -1.17, 1.43  --, -- |
|  | Instructional support^a^ | 5 | -0.00 | (0.00) | -0.00 | (1.63) | -0.02, 0.02 |
|  | Global score^a^ | 3 | -0.04 | (0.06) | -0.79 | (1.00) | -0.77, 0.68 |
| Language/Literacy | Emotional support | 13 | -0.01  -0.01 | (0.00)  (0.00) | -8.28  -6.91 | (1.12)  (1.22) | -0.02, 0.00  -0.01, 0.00 |
|  | Instructional support^a^ | 13 | 0.00 | (0.01) | 0.21 | (1.30) | -0.09, 0.10 |
|  | Managerial quality^a^ | 6 | -0.01 | (0.04) | -0.22 | (1.58) | -0.26, 0.24 |
|  | Global score^a^ | 5 | 0.04 | (0.01) | 3.75 | (1.17) | -0.06, 0.14 |
| Behavioral skills | Emotional support^a^ | 4 | -0.10 | (0.39) | -0.26 | (1.94) | -1.82, 1.62 |
|  | Instructional support^a^ | 5 | 0.00 | (0.02) | 0.03 | (1.11) | -0.20, 0.21 |
|  | Managerial quality^a^ | 3 | -0.30 | (0.55) | -0.54 | (1.00) | -7.27, 6.68 |
| Social competence | Emotional support | 8 | -0.01  -0.01 | (0.01)  (0.01) | -1.71  -1.25 | (2.12)  (1.78) | -0.05, 0.02  -0.07, 0.04 |
|  | Instructional support^a^ | 4 | -0.06 | (0.02) | -3.41 | (1.72) | -0.14, 0.03 |
|  | Managerial quality | 4 | -0.02  -1.28 | (0.06)  (0.00) | -0.34  -0.00 | (1.85)  (1.00) | -0.28, 0.24  --, -- |
|  | Global score^a^ | 3 | -0.16 | (0.02) | -0.00 | (1.00) | -0.38, 0.07 |
| Behavioral problems | Emotional support^a^ | 8 | -0.03 | (0.04) | -0.76 | (2.31) | -0.20, 0.13 |
|  | Instructional support^a^ | 5 | -0.07 | (0.15) | -0.43 | (2.22) | -0.66, 0,52 |
|  | Managerial quality^a^ | 5 | 0.13 | (0.05) | 2.69 | (2.18) | -0.06, 0,32 |
|  | Conflict/Negative emotional climate^a^ | 2 | -0.03 | (0.00) | -- | (--) | --, -- |
|  | Global score^a^ | 3 | 0.11 | (0.01) | 7.62 | (1.00) | -0.07, 0.29 |

*Note*. The statistics reported in the first row for each outcome reflect results without control variables; the statistics reported in the second row for each outcome reflect results with control variables (sex composition: proportion of girls in the sample and average child age in the sample, in months).

^a^Information regarding control variables was not available.
