# Supplementary material for: Early childhood education and care quality and associations with child outcomes: A meta-analysis
Source: PLoS One. 2023 May 25;18(5):e0285985. doi: 10.1371/journal.pone.0285985 (PMC10212181; doi:10.1371/journal.pone.0285985)
Supplement: S11 File — (DOCX) [file pone.0285985.s013.docx]

Early Childhood Education and Care Quality and Associations with Child Outcomes: A Meta-Analysis

Supporting Information (SI) 11

Differences by the Timing of the Data Collection


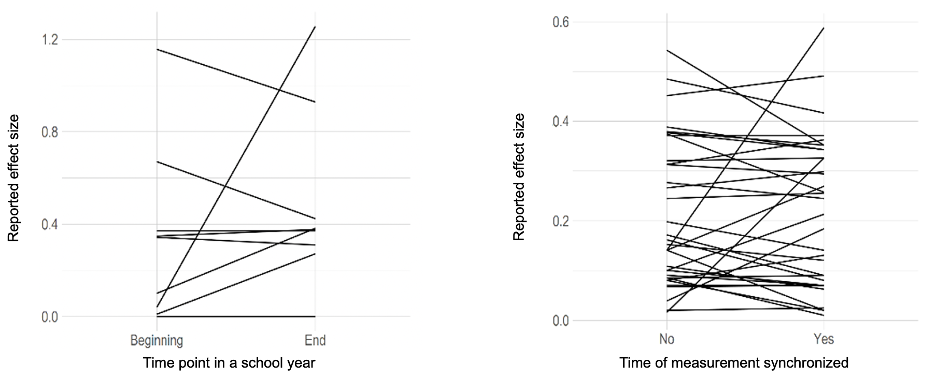


*Figure S4.* Left panel: Comparison of effect sizes based on the time in a school year when the measures were assessed (beginning versus end of the school year). Right panel: Comparison of effect sizes based on the time points within a school year when measures were assessed (quality and outcomes were assessed at the same time point [concurrently] or at different time points).
