# Supplementary material for: Early childhood education and care quality and associations with child outcomes: A meta-analysis
Source: PLoS One. 2023 May 25;18(5):e0285985. doi: 10.1371/journal.pone.0285985 (PMC10212181; doi:10.1371/journal.pone.0285985)
Supplement: S12 File — (DOCX) [file pone.0285985.s014.docx]

Early Childhood Education and Care Quality and Associations with Child Outcomes: A Meta-Analysis

Supporting Information (SI) 12

Differences by Ethnic Minority or Socioeconomic Family Background


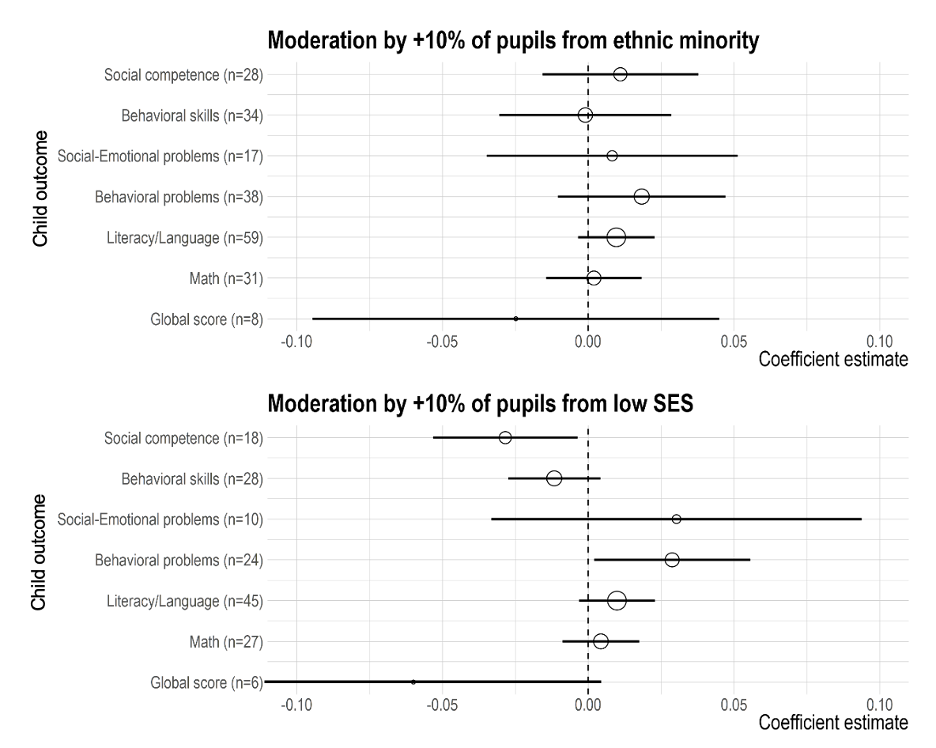


*Figure S5*. Associations between ECEC quality and child outcomes, moderated by family background differences (number of unique studies is given in the parentheses). The estimates’ 95% confidence intervals are shown in lines. Models did not include control variables.

The coefficients can be interpreted as the extent to which the association between ECEC quality and child outcomes changes if the percentage of children from an ethnic minority or a low-income family background increases by 10%.
